# Exploration into the Needs and Requirements of the Remote Driver When Teleoperating the 5G-Enabled Level 4 Automated Vehicle in the Real World—A Case Study of 5G Connected and Automated Logistics

**DOI:** 10.3390/s23020820

**Published:** 2023-01-10

**Authors:** Shuo Li, Yanghanzi Zhang, Simon Edwards, Philip T. Blythe

**Affiliations:** School of Engineering, Newcastle University, Cassie Building, Claremont Road, Newcastle upon Tyne NE1 7RU, UK

**Keywords:** vehicle automation, Level 4 automated vehicles, 5G, teleoperation, remote drivers, real world testing of automated vehicles, qualitative study, user-centred design, user requirements, human–machine interaction

## Abstract

Connected and automated vehicles have the potential to deliver significant environmental, safety, economic and social benefits. The key advancement for automated vehicles with higher levels of automation (SAE Level 4 and over) is fail-operational. One possible solution for the failsafe mode of automated vehicles is a 5G-enabled teleoperation system controlled by remote drivers. However, knowledge is missing regarding understanding of the human–machine interaction in teleoperation from the perspective of remote drivers. To address this research gap, this study qualitatively investigated the acceptance, attitudes, needs and requirements of remote drivers when teleoperating a 5G-enabled Level 4 automated vehicle (5G L4 AV) in the real world. The results showed that remote drivers are positive towards the 5G L4 AV. They would like to constantly monitor the driving when they are not controlling the vehicle remotely. Improving their field of vision for driving and enhancing the perception of physical motion feedback are the two key supports required by remote drivers in 5G L4 AVs. The knowledge gained in this study provides new insights into facilitating the design and development of safe, effective and user-friendly teleoperation systems in vehicle automation.

## 1. Introduction

For the past several years, connected and automated vehicle technology and systems have become a rapidly growing focus of government-funded research and development activities in the field of intelligent transportation systems [1]. Connected and automated vehicles have been widely recognized as potentially able to deliver significant benefits to society; they could greatly improve fuel efficiency and reduce emission, which has the potential to not only significantly contribute to environmental sustainability, but also to economic improvement and transportation decarbonization [2,3]. They could also significantly enhance road safety by potentially preventing traffic crashes caused by human error [4]. In addition, connected and automated vehicles could also play an essential role in reducing traffic congestion by significantly decreasing the average trip times in heavy traffic conditions [5]. Moreover, they also have a strong association with improvements in social inclusion and enhancing accessibility, especially by providing tailored support to user groups with different mobility needs [6,7,8].

Vehicles equipped with connected and automated driving technologies can be grouped into several categories according to system functionalities, as well as the level of human input needed. One of the most commonly adopted classifications is the six-level definition of vehicle automation by SAE International (Society of Automotive Engineers) [9]. Systems of the lower levels of vehicle automation (SAE Level 0 to Level 2) provide different levels of automated features and support the driver in different ways, from providing in-vehicle sensory and informational assistance to completely releasing the driver from the physical driving loop. However, they provide support under one common condition—that the human driver must always be mentally engaged in the vehicle control loop and constantly monitor the driving [9,10]. Compared to the lower levels of automation systems, Level 3 automation systems are the intermediate stage and potentially provide the driver with higher levels of autonomy. This works by enabling them to be temporarily disengaged from driving both physically and mentally and allowing them to safely engage in other non-driving related activities that could be prohibited when driving conventional vehicles, such as using mobile phones or watching movies [1,6,7,11]. However, the biggest challenge for a Level 3 automation system is that it relies on the human driver on-board to reassume control of the vehicle within the provided lead time when the automation system reaches the limitation of its operational design domains [1,9,10]. There could be severe consequences for Level 3 automation systems if the driver fails to take over control of the vehicle effectively within the allocated time frame. Thus, the transition control between Level 3 automation systems and the human driver on-board has become a popular topic for research [10,12,13,14]. For Level 4 automation systems upwards, the system needs to be fail-operational, which ensures the full or degraded operation of the vehicle even if a failure or system limitation occurs, including situations where a human driver fails to take over control when requested [9,10,15]. The failsafe mode has become one of the important challenges for the development of the Level 4 automated systems, which triggers the necessity to explore safe, effective and reliable fail-operational measures. One potential interim solution for the failsafe of Level 4 automated vehicles is a teleoperation system controlled by a remote driver [16]. To enable the teleoperation solution for automated vehicles, 5G technologies play an imperative role, as they can profoundly enhance network connection and provide ultra-low latency communications [17].

The 5G-enabled Connected and Automated Logistic project (5G CAL)cost GBP 4.9M, including GBP 2.4M from 5G Create, an open competition and part of the UK government’s 5G testbeds and trials programme (5GTT) from the UK Department for Digital, Culture, Media and Sport (DCMS). The 5G CAL project designed, developed, deployed and evaluated the 5G-enabled Level 4 automated vehicles (5G L4 AV) with a teleoperation system. The project applied the 5G L4 AV in the context of the logistic sector. The key focuses of the project included: to demonstrate the capability of 5G technologies in enabling safe, effective and secure operation, and the transition of control from the automated vehicle to the teleoperation system; to use 5G technologies to monitor the status of automated vehicles using real-time telematics; and to facilitate understanding of the deployment of 5G-enabled connected and automated logistics at scale. The key members of the project team included Newcastle University, StreetDrone, Vantec, North East Automotive Alliance, Sunderland City Council, Perform Green, Coventry University, Connected Places Catapult, with Nissan, Terberg and Fergusson as contributors.

### 1.1. State of the Art and Research Gaps

Existing research has attempted to explore the teleoperation of vehicle automation from different perspectives. Goodall [18] reviewed the legal environment of the teleoperation of vehicles and developed a model to predict the required number of remote drivers for operating large-scale automated vehicle fleets. The model prediction results indicated that a high proportion of jobs currently belonging to professional human drivers in the USA could potentially be replaced by automated vehicles managed by remote drivers. The research highlighted the importance for the government of reviewing existing policy and paying more attention to teleoperation, which has the potential to significantly facilitate the development of vehicle automation. Some research focuses on the human–machine interface (HMI) of the teleoperation of automated vehicles. Kettwich et al. [16] evaluated a prototype human–machine interface design consisting of video screens, a details screen, disturbances screen, map screen and touchscreen for the teleoperation of SAE Level 4 automated vehicles with thirteen end-users from public transport control centres in Germany. The prototype HMI has received positive feedback from the end-users. Other research focuses on the classification and definition of teleoperation concepts and terminologies. Majstorovic et al. [19] conducted a systematic review on different teleoperation concepts that are used as fallback solutions to deal with critical situations and operation design domain limitations in automated vehicles. Teleoperation concepts were grouped into six categories, including Direct Control, Shared Control, Trajectory Guidance, Waypoint Guidance, Interactive Path Planning and Perception Modification. Moreover, Bogdoll et al. [20] conducted a survey on the terminologies of remote human input systems for automated driving and proposed a taxonomy which potentially provides clarity and reduces confusion in the field of the teleoperation of vehicle automation. Finally, Zulqarnain et al. [21] proposed algorithms to solve the largest challenge of teleoperation systems in automated vehicles—that is, the latency between the teleportation workstation where the remote drivers are located and the automated vehicles. Their research potentially enlightens the selection of suitable locations for teleoperation workstations.

The existing literature and previous studies have researched the teleoperation or remote operation of automated vehicles from various perspectives, including its legal environment and its implications on existing driver jobs, designing and evaluating the HMI and algorithms, as well as defining and classifying terminologies and concepts. However, there are still significant research gaps. For example:

- There is limited research regarding the deployment and evaluation of full-scale authentic automated vehicles incorporating a teleoperation solution in the real world.
- Existing research regarding the teleoperation or remote operation of vehicle automaton has neglected one of the most essential elements of the teleoperation system—the remote drivers (operators).
- Understanding the acceptance, perception and requirements of support from the remote driver’s perspective is important to design and develop safe, effective and user-friendly teleoperation systems for automated vehicles. However, knowledge regarding the needs and requirements of remote drivers when interacting with automated vehicles is limited.

The lack of the above knowledge could potentially thwart the development and real -world deployment of automation systems, especially those of higher levels (SAE Level 4 and over), thereby preventing automated vehicles from delivering the expected environmental, economic, safety and social benefits.

### 1.2. Purpose of the Research

To address the research gaps identified above, the overall aim of this study was to provide new knowledge and unique insights into the teleoperation of vehicle automation by qualitatively investigating remote drivers’ perceptions, attitudes, needs and requirements when teleoperating 5G-enabled Level 4 Automated Vehicles in the real world.

## 2. Materials and Methods

### 2.1. The 5G-Enabled Level 4 Automated Vehicle

**Figure 1:**
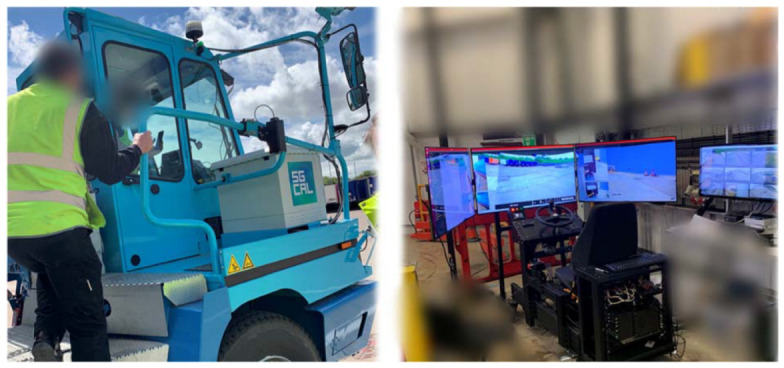
The Terberg autonomous heavy goods vehicle (Left) and the 5G-enabled teleoperation workstation.

This study focused on the 5G-enabled Level 4 Automated Vehicle (5G L4 AV) designed and developed by StreetDrone, a UK-based automated vehicle company. The 5G L4 AV was developed and deployed as a retro-fit on an existing Terberg electric tractor unit as a proof-of-concept project (DCMS 5G Connected and Automated Logistics), with the ambition to trial and demonstrate operational 5G-enabled autonomous delivery utilizing a 40-tonne truck between Vantec UK and Nissan Motor Manufacturing UK in the northeast of England. The 5G L4 AV mainly consists of a converted Terberg electric heavy goods vehicle (HGV) and a 5G-enabled teleoperation workstation. The system is powered by the 5G technology application (Ofcom shared access spectrum N77). Figure 1 shows the 5G L4 AV and the teleoperation station.

### 2.2. Participants

The participants of this study were recruited using the following criteria: they were required to have valid UK driving licenses; be active drivers; and have experience of and be actively engaged with the 5G-enabled teleoperation system of the 5G L4 AV employed in this study, in order to participate. The sample recruitment resulted in six remote drivers.

### 2.3. Study Design

**Figure 2:**
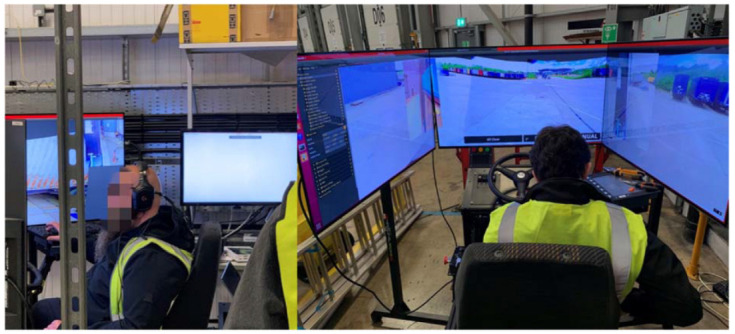
Remote drivers operating the 5G-enabled Level 4 automated vehicle via 5G enabled teleoperation system.

The 5G-enabled L4 AV designed and developed in the 5G CAL project enables automated driving classified as SAE Level 4 automation (SAE J3016). As defined by SAE, the Level 4 automation system refers to a system where an automated driving system responds to all aspects of the dynamic driving task, even if a human driver does not respond appropriately to a request to intervene [9]. One important safety use case of the 5G CAL is the transition of the control process of the automated vehicle during critical situations that fall outside of the capability of the automation system. In such critical situations, the automated driving system will pull to a stop and activate the ‘fail-safe’ mode, where the control of the vehicle will be taken over by the teleoperation system powered by the 5G network connection and controlled by a specially trained human remote driver. Therefore, the use cases concerned in this study mainly focus on the interaction between the three parties during a transition of control process in the L4 AV: the automated driving system, the teleoperation system controlled by the remote driver and a human on-board safety driver. Figure 2 shows a remote driver remotely controlling the 5G L4 AV.

### 2.4. Qualitative Data Collection

Corresponding to the aim of this study, the nature of this research is qualitative. To collect qualitative data, a semi-structured interview was used. This is a proven and effective method in gathering user requirements [22,23,24]. The structured part of the semi-structured interview consisted of seven topics, as follows:

- What is your general opinion of the Level 4 automated vehicle?
- If you are sitting on the remote-control workstation and the vehicle is performing automated driving, what would you do?
- How would you prefer to be informed about a remote-control request of the automated vehicle?
- What are the differences and similarities between operating the vehicle on-board and remotely?
- When performing the remote control of the automated vehicle, what difficulties have you encountered?
- When you are performing the remote control of the automated vehicle, what support do you need?
- Do you have any recommendations to the original equipment manufacturers (in terms of vehicle design and remote-control workstation design)?

### 2.5. Research Process

**Figure 3:**
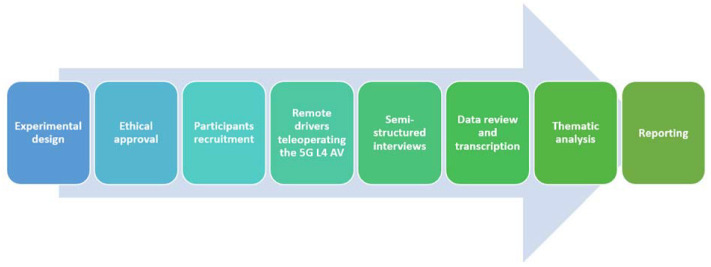
Illustration of research process.

The research process is illustrated in Figure 3. The first step was to develop the qualitative experimental design and then submit it to Newcastle University’s Ethical Committee for review. After ethical approval was granted, the research team contacted the subjects eligible for this research within the project. Then, the qualitative data collection started. Before taking part in the semi-structured interview, all participants were actively engaged with the teleoperation system of the 5G L4 AV. Then, the remote drivers’ opinions, perceptions, needs and requirements were explored and investigated using the semi-structured interview. The interviews were executed using virtual meeting software and via mobile phone. After that, the interviews were transcribed by the research team. The transcribed data were reviewed against the original interview by two researchers separately. Then, the data were analysed using a thematic analysis consisting of six key steps, including data familiarization, coding data, searching, reviewing and defining themes, as well as reporting [22,25]. Then, the identified core themes were interpreted in light of the existing knowledge and literature. Finally, the findings were reported.

## 3. Results and Discussion

### 3.1. Summary of the Thematic Analysis

**Figure 4:**
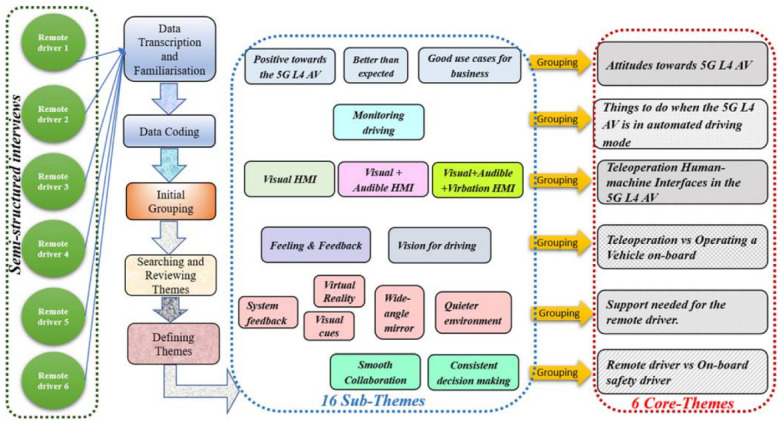
Illustration of thematic analysis of remote drivers’ needs and requirements towards the 5G L4 AV.

As illustrated in Figure 4, the thematic analysis initially yielded sixteen themes, and these were further grouped into six core themes representing remote drivers’ perception, needs and requirements towards the 5G L4 AV. The attitudes were towards the 5G L4 AV; things to do when the 5G L4 AV is in automated driving mode; teleoperation human–machine interfaces in the 5G L4 AV; teleoperation vs. operating a vehicle on-board; support needed for the remote driver; and remote driver vs. on-board driver. The core themes are presented in the following sections.

### 3.2. Theme 1: Attitudes towards the 5G L4 AV

The first theme regards the remote drivers’ overall perception and attitudes towards the 5G L4 AV following the interaction and teleoperation of the system in the real world. As shown in Table 1, the first theme consists of three sub-themes—‘Positive towards the 5G L4 AV’, ‘Better than expected’ and ‘Good use cases for business’.

In general, the remote drivers were positive towards the 5G L4 AV and believed the interaction to be better than their expectations. This could be because this is one of the world’s first pieces of research demonstrating the application of the 5G L4 AV in the field of logistics in the real world. Further, the key features of the 5G network, including low latency and fast speed, enabled the 5G L4 AV deployed in this proof-of-concept project to be able to perform effectively and smoothly, which led to positive perceptions among the participants. Another explanation could be that the remote drivers were part of the control loop of the 5G L4 AV deployed in this study, and they were actively engaged and interacted with the system. The actual interaction and hands-on experience could be associated with positive perceptions towards automated vehicles [23,26]. The remote drivers also perceived that the 5G L4 AV is good for business. A possible explanation could be that this study was conducted as part of the 5G CAL project exploring the application of the 5G L4 AV in logistics. Some remote drivers in this study were logistics drivers who were familiar with the use case, which may have partially enriched their understanding of the potential benefits of the 5G L4 AV for business.

### 3.3. Theme 2: Things to Do When the 5G L4 AV Is in Automated Driving Mode

The second theme explores the non-driving related tasks that remote drivers prefer to perform when the 5G L4 AV is operating in automated driving mode. The example quotes are displayed in Table 2. The remote drivers’ responses were consistent in that they would like to keep their eyes on the driving environment even when they are not operating the vehicle remotely. This finding is different from previous research exploring the type of non-driving related tasks in automated vehicles where the end-users exhibited a preference of conducting a wide range of non-driving related activities in automated vehicles, including those they are not allowed to perform when operating a conventional vehicle, such as reading and relaxing [23]. This could be partly due to the fact that this was the first time for the remote drivers in this study to actually interact with an L4 AV in real life. Therefore, they were very cautious and careful even if they were not operating the system. Another important reason could be that the role of the remote drivers of the 5G L4 AV is different in nature compared to the end-users of passenger automated vehicles. They are not only one party of the control loop of the 5G L4 AV, but also fundamental to the role of failsafe in the system. Therefore, they perceived that they would need to be constantly monitoring the system. However, monitoring the 5G L4 AV driving on the teleoperation workstation does not require the same level of physical and cognitive workload from the drivers as driving a conventional vehicle manually. Such cognitive underload could lead to deteriorated performance, passive fatigue and inattention among the remote drivers [27,28]. To date, the regulation and legislation regarding permitted non-driving activities among the remote drivers of automated vehicles is limited. The findings of this study suggested that future work should investigate and quantify remote drivers’ performance, attention and workload to ensure the safe and efficient teleoperation of the 5G L4 AV.

### 3.4. Theme 3: Teleoperation Human–Machine Interfaces in the 5G L4 AV

The third theme regards the remote drivers’ preferences towards the modality of the human–machine interfaces of the teleoperation system. As shown in Table 3, this theme consists of three sub-themes representing three different modalities of HMI. Remote drivers indicated that they prefer to be informed about a critical situation using the visual combined with audible HMI. This is in accordance with the findings of previous research suggesting that using visual and auditory HMI is beneficial to user performance [11,29,30]. Some remote drivers also mentioned that visual and audible combined with vibration in the HMI could be potentially useful. Despite teleoperation by the remote drivers forming one part of the control loop for the 5G L4 AV, remote drivers are not physically within the actual driving environment of the 5G L4 AV, so the vibration element of the HMI could become an additional stimulus that ensures the remote driver focuses on the critical situation more quickly and effectively. Future work should be conducted to integrate remote drivers’ needs into the design of the HMI of the teleoperation system, and then to evaluate the usability of the design.

### 3.5. Theme 4: Teleoperation vs. Operating a Vehicle On-Board

The fourth theme focuses on the remote drivers’ perception of the control of the 5G L4 AV remotely via the teleoperation system, compared to manually driving a conventional vehicle. It consists of two sub-themes—feeling and feedback, and vision, for driving. Example quotes are shown in Table 4. The participants indicated that operating the 5G L4 AV remotely via the teleoperation system felt somewhat different to operating a conventional vehicle as an on-board driver. The differences mainly concerned the feeling of operating the vehicular controls (steering wheel, acceleration and brake pedals), as well as the feedback from the vehicle and the driving environment. In addition, remote drivers also felt that the field of vision for driving when teleoperating the 5G L4 AV remotely was slightly limited compared to manually driving a conventional vehicle. The possible explanation could be that the teleoperation system of the 5G L4 AV in this study was developed using a fix-based workstation without a dynamic physical feedback motion system. It has three screens which provide an approximately 180-degree view. These construction features may have reduced the perceptual fidelity and motion fidelity of the teleoperation system [31,32], which could lead to different physical experiences for the remote drivers compared to manual driving. This is an important implication in terms of the physical construction and hardware of the teleoperation system of 5G L4 AVs. To ensure the fidelity of the tele-operation control, especially from the perceptual and motion dimension, future design should include a 360-degree field view and full-feedback motion system [32].

### 3.6. Theme 5: Support Needed for the Remote Driver

The fifth theme concerns the remote drivers’ requirements in terms of support needed when teleoperating the 5G L4 AV. It consists of five sub-themes, including system feedback, virtual reality, visual cues, wider angle mirror and quieter environment. Example quotes are displayed in Table 5.

The remote drivers’ support needs correspond to differences in physical experience discussed in Theme 4. Specifically, remote drivers would like to have more system feedback. As discussed in the previous section, this requirement could be fulfilled by introducing a full-feedback motion system. Moreover, remote drivers believed that using Virtual Reality (VR) would potentially benefit their performance by enhancing peripheral vision. This has raised a strong research need regarding the potential of applying VR in the teleoperation system of 5G L4 AVs. The virtual presence for the users created by VR could enable them to feel that they are physically in a location or a space [33], which, in the context of teleoperation, could potentially increase several dimensions of fidelity, including behavioural fidelity, task fidelity and perceptual fidelity [32]. Moreover, remote drivers believed that visual cues, a wide-angle mirror and a quieter environment (the workstation was located in an active warehouse) would benefit their performance. These measures could enhance the fidelity of the teleoperation from the perceptual dimension.

### 3.7. Theme 6: Remote Driver vs. On-Board Safety Driver

The last theme describes remote drivers’ perception in terms of collaborating with the on-board safety driver in the 5G L4 AV. It consists of two sub-themes—smooth collaboration and consistent decision making. Example quotes are shown in Table 6. The nature of the 5G CAL project is proof of concept. When the 5G CAL was being developed and deployed, the on-board safety driver was responsible for operating the vehicle in some situations, such as loss of 5G connection. The remote drivers believed that their collaboration with the on-board safety drivers was smooth, and they were in constant communication if there was any issue. They exhibited consistent decision making in terms of whether the vehicle should perform a ‘GO’ or ‘NO GO’ command. These findings have important implications. In this study, the use case was 5G Connected and Automated Logistics and the person on-board was the safety driver. For other use cases of the 5G L4 AV, the people on-board could be passengers. The findings of this study indicated that constant and clear communications between the remote driver and the people on-board could potentially facilitate the usability of the entire system. Future work should be conducted to investigate this. Moreover, while the findings from the current study showed that decision making was consistent between the remote driver and the on-board safety driver, further research should assess whether there is any difference between the two parties from a performance perspective: for example, the time-aspect of the takeover as well as the quality of takeover control [10].

## 4. Conclusions and Future Work

Connected and automated vehicles have the potential to deliver great benefits in terms of enhancing road safety, reducing traffic emission, optimising road efficiency and improving social inclusion [23]. They also potentially bring new opportunities to fundamentally change urban mobility and logistics services [34]. Among the vehicles equipped with higher levels of automation systems (SAE Level 3 and over), Level 4 automated vehicles could include a failsafe mode which ensures the safety of the vehicle in urgent situations where the vehicle is out of the designed service areas [9,10]. The failsafe mode could be achieved by a teleoperation system in which a human driver takes over control and operates the vehicle remotely [16]. However, to date, limited research has explored the perception, attitudes, needs and requirements of the remote drivers of Level 4 automated vehicles. To fill this research gap, this research used a qualitative methodology to investigate the remote drivers’ perception, needs and requirements when operating the L4 AV remotely via a 5G-enabled teleoperation system in the real world. The study identified six core themes representing the needs and requirements of the remote drivers who perform the essential role of teleoperation control of the system. The research found that the remote drivers exhibited positive attitudes towards the 5G L4 AV. They also highlighted the need for further improvement of the human–machine interaction in the teleoperation system of the 5G L4 AV in terms of the visual field for driving, as well as the perception of feedback from the vehicle. In summary, the main findings of this study are as follows:

- Remote drivers have positive attitudes towards the 5G L4 AV.
- Remote drivers would be monitoring the road when the 5G L4 LV is performing automated driving. They expect to be informed if something happens.
- In terms of the human–machine interface, remote drivers would like to have verbal communication if there is a safety driver on-board. If there were no safety drivers on board (ultimately the desired scenario), a visual, audible and vibrational HMI would be beneficial.
- The main difference and difficulties remote drivers experienced when controlling the vehicle remotely (compared to conventional manual driving) was lack of depth vision, as well as not being able to feel the feedback from the vehicle when executing a manoeuvre.
- Remote drivers would like more support regarding their visual field driving when teleoperating the 5G L4 AV.
- Remote drivers would like more support in terms of enhancing the perception of physical feedback when teleoperating the 5G L4 AV.
- Possible support includes introducing Virtual Reality, wide angle mirrors, as well as full motion feedback systems into the teleoperation workstation of the 5G L4 AV.
- Remote drivers collaborated smoothly with the on-board safety driver, and their strategic decision making in urgent situations was consistent with the on-board safety drivers’.

This study is one of the world’s first pieces of research adopting a qualitative methodology to explore the user requirements from the remote driver perspective of a 5G L4 AV system deployed in the real world. The findings of this work have important implications for stakeholders of the 5G L4 AV, including manufacturers, policymakers, academics and researchers, in terms of the development and deployment of safe and efficient human–machine interactions of teleoperation systems in the 5G L4 AV. This study strengthened the importance of implementing a user-centred approach when designing, developing and deploying future mobility technologies and connected and automated vehicles [11,23,35,36]. The findings and new knowledge gained in this research could be developed and used in the following directions to enlighten future work. To begin with, the remote drivers of the L4 AV perceived some differences between operating the vehicle on board and via the teleoperation system. Therefore, ensuring the fidelity of a teleoperation system in terms of enhancing the remote driver’s sensing (vision and hearing) of the driving environment, as well as improving their perception of physical motion feedback from the vehicle, could be an important challenge for the future development and deployment of L4 AVs. Future work could focus on exploring potential measures to enhance the perceptual fidelity and motion fidelity of the teleoperation workstation of the 5G L4 AV. Apart from that, tailored training could also be potentially useful for remote drivers to adapt the teleoperation system more efficiently and rapidly. Future work could explore what kinds of training are needed for a conventional vehicle driver to become a remote driver and assess the training quality and outcome. In addition, future work could further explore and evaluate which modality of the human–machine interfaces could lead to better performance among remote drivers. This study investigated remote drivers’ requirements when teleoperating the 5G L4 AV with a safety driver on-board. Future work could explore what their needs are when there is no safety driver. Moreover, the study found that remote drivers would like to monitor the vehicle driving when they are not teleoperating the vehicle. In future work, it would be worth seeking to quantify and evaluate remote drivers’ attention, mental workload and situation awareness while monitoring the system. It is also necessary for future work to investigate the potential impact of distraction and driving disengagement [8], as well as performing non-driving related tasks, on remote drivers’ attention and performance. Moreover, this study is from the perspective of the remote driver who is an essential part of the teleoperation process—the failsafe mechanism of the 5G L4 AV. Future work could focus on different stakeholders of 5G L4 AVs, for example, from the perspective of the end-users and customers who are planning to adapt 5G L4 AVs in their service and business. Although the study yielded novel findings and has key implications for future work, there are still some limitations. Firstly, this study is qualitative in nature; it does not aim for the generalizability of the results but to develop contextualized and in-depth knowledge regarding the remote drivers’ interaction with the 5G L4 AV [37]. Future studies could research the teleoperation of the Level 4 AV with a larger sample size and adopt quantitative methodologies to further analyse the findings. The key knowledge gained in this study could facilitate the experimental design. Moreover, future research could explore whether the demo-graphic factors [6,7] of the remote driver would impact their needs and requirements when interacting with the 5G L4 AV. The ultimate goal may be that by understanding these critical interactions better, we will be able to transition to fully driverless L4 vehicles with a remote driver monitoring a number of vehicles. This would deliver a real economic benefit to the operator. Overall, the knowledge gained in this study could be important evidence potentially informing the development of strategy, policy, practice and service provision [38] in the field of 5G-enabled automated vehicles.

## Figures and Tables

**Table 1:** Theme 1: Remote drivers’ attitudes towards the 5G L4 AV.

Theme 1: Attitudes towards the 5G L4 AV	
Sub Themes	Example Quotes
1.1 Positive towards the 5G L4 AV	‘I think obviously it’s looking really good, you can get good feedback from the remote or teleportation side by the end of the project. The visibility and visual aids that the person’s got are really good in terms of mirrors and the replicating what you would get if you’re in the vehicle.’ ‘Like nothing I’d ever seen before. Couldn’t take my eyes off the screen.’ ‘It seems amazing to me.’ ‘Definitely, really enjoyed working with it.’
1.2 Better than expected	‘Quite impressed with it, how it handled (electric) expected it to be more complex but it’s basically ‘click a switch’.’ ‘At first, I could never see it working—thought it was crazy. As time went on I was amazed.’
1.3 Good use cases for business	‘I think it has some very good use cases. I don’t think it’s applicable for public, you know, average consumer. I think it got some good use cases for business in controlled environment.’

**Table 2:** Theme 2: Things to do when the 5G AV is automated driving.

Theme 2: Things to Do When the 5G L4 AV Is Automated Driving	
Sub Themes	Example Quotes
2.1 Monitoring driving	‘Obviously, the screens are displaying the same video data that they always are, so you can kind of keep an eye on what the vehicle is doing, but there’s no role as it were.’ ‘I don’t need to do anything until it tells you there’s a problem. If it’s level 4 I don’t need to constantly be monitoring it, there should be enough time for me to take over rather than an immediate thing. I would do whatever I would.’ ‘Just sit and have a look at the cameras.’ ‘I’m observing it all the time. It can’t check way points by itself. I’m checking way ahead is clear.’ ‘All I do is keep an eye on for any problems.’

**Table 3:** Theme 3: Teleoperation human–machine interfaces in the 5G L4 AV.

Theme 3: Teleoperation Human–Machine Interfaces in the 5G L4 AV	
Sub Themes	Example Quotes
3.1 Visual HMI	‘On a screen. Visual prompt on the screen.’
3.2 Visual + Audible HMI	‘So, at the moment there’s obviously a message popping up bottom left hand corner of the screen. Probably would be beneficial if there’s some kind of audible noise as well. So, I don’t think we have at the moment. In case you’re looking at the way or looking at something different.’ ‘If there’s safety driver in the vehicle, I’ll definitely want a verbal communication with them. Where there’s no safety driver and of course it has to be like a digital like pad in front of you, tells you this is what’s going on.’ ‘The way it was done was mobile phones linked to a headset. There was also a Bluetooth speaker so XXX and the software engineer could hear what was going on. This was fine.’ ‘Just a clear instruction would be nice, don’t know how practical that is on a large scale.’
3.3 Visual + Audible + Vibration HMI	‘Audible is usually good, an audible alert as well as visual, some sort of buzzer maybe not a siren, what could potentially also be useful is like a pack tile. A frequency device that can vibrate the chassis, I feel that would be a very noticeable thing. Even if you’re listening to music or watching a film or something.’

**Table 4:** Theme 4: Teleoperation vs. operating a vehicle on-board.

Theme 4: Teleoperation vs. Operating a Vehicle On-Board	
Sub Themes	Example Quotes
4.1 Feeling and Feedback	‘The similarities are we got quite good views on what’s around you. I think differences are, again, it’s the feel of the vehicle, so acceleration, deceleration, you don’t get any of that feel. And then also the audible kind of feedback of what’s going on at the vehicle, you don’t get any feel of that either.’ ‘The main difference is you can’t feel it. In normal driving you can, like you feel your foot on the pedal and you can like steel the vehicle like rolling forward, you can hear it, you turn the wheel, you can hear it. In the remote driver seat, you don’t get that at all.’ ‘Major difference is lack of feedback from steering wheel, e.g., bumps in the road.’
4.2 Vision for driving	‘Cos obviously you don’t get a size of the vehicle as easily, so it can be more difficult to tell distance, obviously you’re looking at a 2D screen, so you can’t get the 3D without VR or something like that, then you could have the depth vision. But at the moment the way it is currently, it’s hard to have a perception of speed and the size of your vehicle.’ ‘The camera angles, obviously when you’re in a car you’ve got 360 degrees view.’ ‘In a real truck you can turn your head and see behind you—in a tight reverse in remote control you lose the vision of where the rear of the trailer is. Wide angled mirrors can help. This maybe an area for more improvement.’ ‘I think the main difficulties are seeing those kind of blind spots and see kind of right in front of you when it’s really close around you, so kind of sometimes gauging the vehicles, if the road is quite narrow, gauging how close you are on either side is a little difficult because you haven’t got that video right down in front of you. And also I kind of witnessed some of the guys when we done live loads with a heavier trailer, the difference between a light trailer versus a heavy trailer, you don’t necessarily get the feel for that from the remote driver either.’

**Table 5:** Theme 5: Support needed for the remote driver.

Theme 5: Support Needed for the Remote Driver	
Sub Themes	Example Quotes
5.1 System feedback	‘Some extra camera views and then also extra feedback in terms of probably movement of the seat or the wheel and things like that would help. Also, potentially some feedback through the wheel of pedals and you’ve got a different load on.’
5.2 Virtual reality	‘VR could be one option; it could give you the depth perception than you could have your peripheral vision and be able to see things more clearly.’
5.3 Visual cues	‘Visual cues on the monitors could be a good way to have markers for where your vehicle is.’
5.4 Wide angle mirror	‘In a real truck you can turn your head and see behind you—in a tight reverse in remote control you lose the vision of where the rear of the trailer is. Wide angled mirrors can help. This maybe an area for more improvement.’
5.5 Quieter environment	‘Quieter office space rather than warehouse floor.’

**Table 6:** Theme 6: Remote driver vs. on-board safety driver.

Theme 6: Remote Driver vs. On-Board Safety Driver	
Sub Themes	Example Quotes
6.1 Smooth collaboration	‘Remote operators are responsible for some operations, XXX takes over if the system goes down. There were a few instances where the 5G connection was lost. These were teething problems in specific locations—they were resolved.’ ‘Yes, in constant communications. I wouldn’t authorise without his authority first.’
6.2 Consistent decision making	‘I don’t see any occasions whereas a remote driver we’ve been keen to proceed, but then the safe driver put their foot on the brake to stop it effectively. So, I think it’s been really successful in terms of the decision that the remote driver is making.’ ‘Yean, it’s pretty much exact same.’ ‘Yes, my decisions correspond to the safety driver’s. I can see the entire area; they can see like everything.’

## Data Availability

The data that support the findings of the current study are available on request from the corresponding author.
